# Theoretical and Experimental Studies on the Crystal Structure, Electronic Structure and Optical Properties of SmTaO_4_

**DOI:** 10.3390/ma9010055

**Published:** 2016-01-18

**Authors:** Song Wang, Miao Jiang, Lihong Gao, Zhuang Ma, Fuchi Wang

**Affiliations:** School of Materials Science and Engineering, Beijing Institute of Technology, Beijing 100081, China; 2120131177@bit.edu.cn (S.W.); jiangmiao0226@gmail.com (M.J.); hstrong929@bit.edu.cn (Z.M.); wangfuchi@bit.edu.cn (F.W.)

**Keywords:** crystal structure, electronic structure, optical properties, first principles calculation

## Abstract

The crystal structure, electronic structure and optical properties of SmTaO_4_ were identified through an experimental method and first principles calculation. X-ray powder diffraction (XRD) and a spectrophotometer were used to characterize the crystal structure, reflectivity and band gap of this material; furthermore, the electronic structure and optical properties were investigated according to three exchange-correlation potentials, LDA, GGA and GGA + *U*. Results show that the SmTaO_4_ calcined at 1400 °C with the solid-state reaction method is in monoclinic phase in the space group *I*^2^/*a*. In addition, the calculated lattice parameters are consistent with the experimental values. The electron transitions among the O 2*p* states, Sm 4*f* states and Ta 5*d* states play a key role in the dielectric function, refractive index, absorption coefficient and reflectivity of SmTaO_4_. The calculation of first principles provides considerable insight into the relationship between the electronic structure and optical properties of this material.

## 1. Introduction

Lanthanide orthotantalates (LnTaO_4_; Ln = La, Ce, Pr, Nd and Sm, *etc.*) have recently been studied extensively because of their favorable dielectric property [1], luminescence [2,3], chemical and electrochemical stabilities [4], photo-electronic activity [5,6], and ion conductivity [7,8]. LnTaO_4_ can be used in phosphors for solid-state lighting, in photocatalysts for both contaminant degeneration and H_2_ production, in chemically robust hosts for nuclear materials and wastes, in solid-state laser materials, and in ion conductors for lithium batteries or solid-oxide fuel cells [9,10,11,12].

Electronic interactions, including the partly occupied Ln 4*f* levels, play an important role in the properties of LnTaO_4_ [13,14]. Machida *et al.* [15,16] reported that the energy level of Ln 4*f* states can be lowered by increasing the number of 4*f* electrons; thus, the unoccupied 4*f* states are at the bottom of the conduction band, whereas the partially occupied 4*f* states drop with an increase in 4*f* electrons and finally fall into the valence band. Given that Sm is located in the middle position in the lanthanide series and that it contains six 4*f* electrons, the Sm 4*f* states may be located near the Fermi level, thereby increasing the probability that electron transition is enhanced. This phenomenon satisfies the requirements of high reflectivity, *i.e.*, the band gap is narrow, the conduction band moves to a low level energy position, and the Fermi level is located in the conduction band [17]. Therefore, SmTaO_4_ may exhibit excellent optical properties and may be applied in protective or anti-radiation materials for optical use.

To our knowledge, a few structural and luminescent investigations have been conducted on SmTaO_4_ [3,4,7], while its electronic structure and optical properties have rarely been reported. In this paper, SmTaO_4_ was successfully synthesized through the solid-state reaction method, and its electronic structure and optical properties were investigated in detail. Its electronic structure and optical properties are correlated through first principles calculation.

## 2. Experimental Methods and Theoretical Calculations

### 2.1. Experimental Methods

Analytical-grade Ta_2_O_5_ and Sm_2_O_3_ powders were utilized as raw materials. The raw powders were preheated at 800 °C to remove the absorbed moisture and other gases; subsequently, these powders were weighed at a 1:1 stoichiometric ratio and then milled for 6 h in a ball-grinding mill with alcohol as a medium. The powders were then calcined at 1400 °C for 10 h. The phase composition of SmTaO_4_ was determined through X-ray powder diffraction (XRD, X’Pert PRO MPD, PANalytical Inc., Almelo, The Netherlands) with monochromated CuKα radiation and a scintillation detector at a scanning rate of 4°·min^−1^. The experimental values of lattice constants, unit cell volume, interatomic distances and atomic coordinates were calculated using Reflex Plus software packed in MS Modeling (Accelerys Inc., San Diego, CA, USA) with Rietveld refinement [18]. In addition, the calculated XRD pattern of structure model was also obtained via Reflex Plus. The reflectivity was measured by a spectrophotometer (Cary 5000, Varian Inc., Palo Alto, CA, USA) operating in the UV/VIS/NIR spectral range with a 110 mm integrating sphere. The band gap was determined from the diffuse reflectance spectrum as follows [19,20]:

Step 1. The relationship between band gap (*E_g_*) and photon frequency is described in the Tauc equation:

(1) (hνα)1n=A(hν−Eg)

where *h* is the Planck’s constant, *ν* is the photon frequency, α is the absorption coefficient, *E_g_* is the band gap, and *A* is the proportional constant. The value of exponent *n* denotes the nature of sample transition; *n* = 1/2 for direct allowed transitions and *n* = 3/2 for direct forbidden transitions. Moreover, *n* = 2 for indirect allowed transitions and *n* = 3 for indirect forbidden transitions. Given that direct allowed transitions are considered in this study, *n* = 1/2 is chosen for this sample.

Step 2. The acquired diffuse reflectance spectrum is converted into a Kubelka–Munk function and the vertical axis is converted into quantity *F*(*R*∞), which is proportional to the absorption coefficient. Thus, α in Equation (1) is substituted with *F*(*R*∞) as follows:

(2) $${\left(h\nu F\left(R\infty \right)\right)}^{2}=A\left(h\nu -E_{g}\right)$$

Step 3. When the Kubelka–Munk function is employed, the (*hνF*(*R*∞))^2^ is plotted against *hν*. *hν* is expressed in electron volts (eV), and its relationship with wavelength λ (nm) is written as *hν* = 1239.7/λ.

Step 4. A line is drawn tangent to the inflection point on the curve obtained in Step 3, and the *hν* value at the intersection point of the tangent line and the horizontal axis corresponds to the band gap *E_g_* value.

### 2.2. Computational Details

The theoretical calculations were performed with the pseudo-potential plane-wave total energy implemented in the Cambridge Sequential Total Energy Package code (CASTEP code). The interactions between electrons and ion cores were represented by the Vanderbilt-type ultra-soft pseudo-potential. Furthermore, the O 2*s*^2^, 2*p*^4^ electrons, Ta 5*d*^3^, 6*s*^2^ electrons and Sm 5*s*^2^, 5*p*^6^, 4*f*^6^, 6*s*^2^ electrons were explicitly regarded as valence electrons. The standard density functions describe poorly the electronic properties of these localized orbitals, but they perform very well in the total energy calculation which refers to structural properties. So to calculate the crystal structure, the exchange-correlation effects were described by the conventional local density approximation (LDA) of Ceperley-Alder-Perdew-Zunger (CA-PZ) [21] and generalized gradient approximation (GGA) of Perdew-Burke-Ernzerhof formula (PBE) [22]. To compute the electronic structure and optical properties, the GGA with additional Hubbard correlation terms, namely the GGA + *U* approach (where *U* is the Hubbard energy) [23,24] was applied in addition to LDA and GGA because the GGA + *U* approach effectively describes the on-site electron-electron repulsion associated with the 4*f* narrow bands of lanthanide atoms [25]. Hubbard U was set as 6.0 eV for the valence *f* shell of Sm atom which has already been successfully applied in lanthanide compounds [25,26,27]. A plane-wave cut-off energy of 600 eV was employed throughout the calculation process. To sample the Brillouin zone (BZ), the following *k*-point grid calculations were generated according to the Monkhorst–Pack scheme: 5 × 5 × 5 for structure optimization and the band structure [28]. In order to improve the calculation precision of properties, the k-point grids are promoted to 6 × 6 × 6 for electronic density of states and 8 × 8 × 8 for optical properties. Moreover, the Pulay scheme of density mixing [29] was applied to evaluate energy and stress levels. The Broyden-Fletcher-Goldfarb-Shanno (BFGS) minimization scheme [30] was also applied in geometry optimization. The tolerances for energy change, maximum force, maximum stress, and maximum displacement were set as 2 × 10^−5^ eV atom^−1^, 0.05 eV Å^−1^, 0.1 GPa, and 0.002 Å, respectively. The total energy converged to 1 × 10^−6^ eV atom^−1^ in the self-consistent calculation process.

## 3. Results and Discussion

### 3.1. Crystal Structure

**Figure 1:**
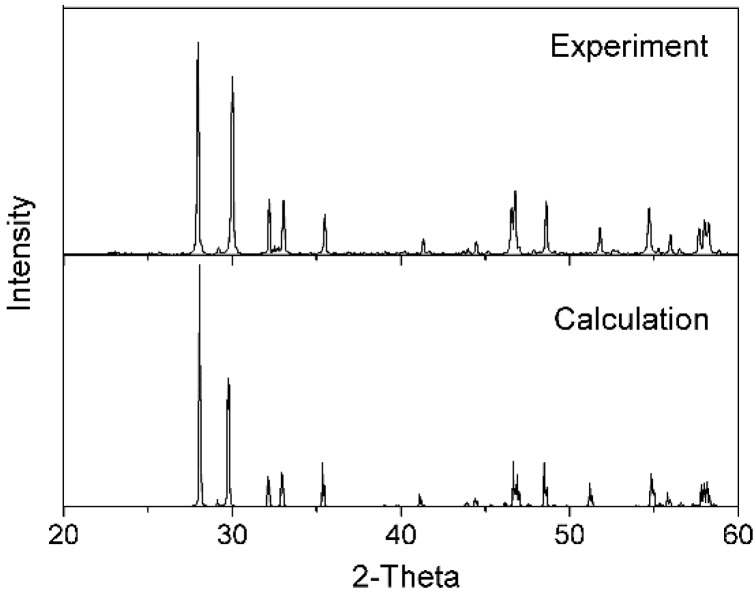
X-ray diffraction patterns of SmTaO_4_: (**a**) Experimental result; (**b**) Calculated result.

Figure 1 shows the XRD pattern of the sample calcined at 1400 °C. All of the peaks match those of the SmTaO_4_ phase. The SmTaO_4_ crystal structure belongs to the monoclinic system, and the coincident space group is *I*^2^/*a*. The Sm^3+^ ion is surrounded by eight oxygen neighbors to form a dodecahedron and is located at the sites with C_2_ symmetry; moreover, the Ta^5+^ ion is surrounded by six oxygen neighbors to form a distorted octahedron [31]. The experimental SmTaO_4_ crystal structure is refined according to the XRD result using Reflex Plus software, and this refined structure is adopted as the structure model for use in theoretical calculation.

**Figure 2:**
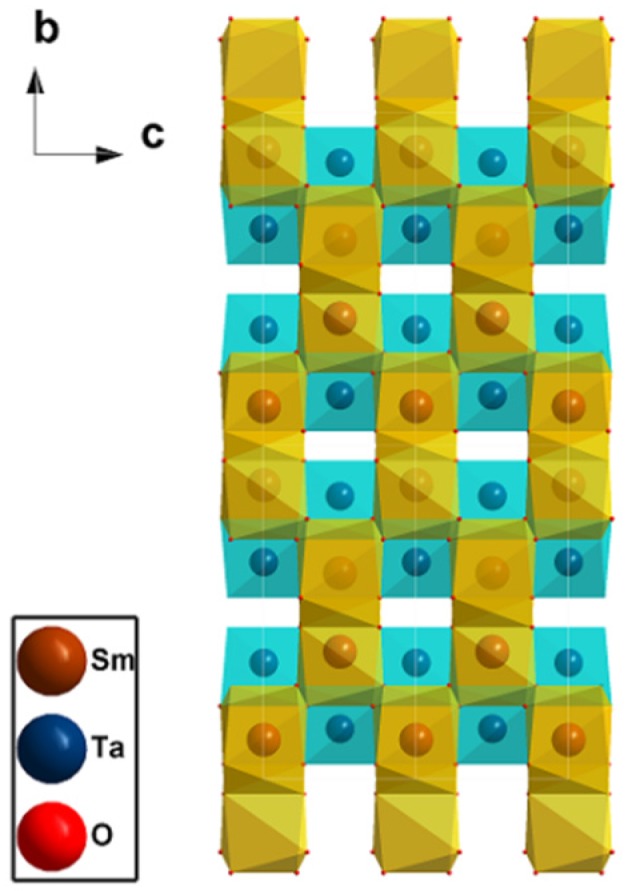
Crystal structure of SmTaO_4_.

Figure 2 depicts the refined crystal structure. The SmO_8_ dodecahedron is generated, along with its two neighbors, along the *b*–*c* plane to form a general folding line shape, and the TaO_6_ octahedrons connect these dodecahedrons with their vertices. Figure 1b presents the corresponding XRD pattern calculated based on this crystal structure; this result matches the experimental outcome, and this finding verifies the accuracy of the structure model.

The theoretical SmTaO_4_ structure was investigated with both LDA and GGA exchange-correlation functions. Table 1 presents the experimental, calculated, and reported lattice constants (*a*, *b*, *c*, and β), the conventional cell volume (*V*), and the density of SmTaO_4_. Table 2 and Table 3 list the calculated and experimental interatomic distances and atomic coordinates of SmTaO_4_ compared with reported results The computed findings show that the approximate density functional theory (DFT) executed by either LDA or GGA underestimated or overestimated the lattice constants and volume slightly more than the experimental values. Nonetheless, the calculated lattice constants differ from the experimental values by no more than 1.16%. Additionally, the calculated interatomic distances and atomic coordinates are very close to the experimental ones. So there is a good agreement between our experimental and calculated results, and all of these values match well with the available reported values. This agreement between calculation and experiment ensures the accuracy of the structure model used to derive physical properties.

**Table 1:** Experimental, calculated and reported lattice constants (*a*, *b*, *c*, and β), conventional cell volume (*V*), and density of SmTaO_4_.

Lattice Constant	Experimental	LDA	GGA	Reported
*a* (Å)	5.444 (±0.0085)	5.393	5.514	5.455 ^a^, 5.405 ^b^
*b* (Å)	11.115 (±0.0072)	10.968	11.241	11.137 ^a^, 11.063 ^b^
*c* (Å)	5.081 (±0.0079)	5.094	5.161	5.091 ^a^, 5.081 ^b^
*β* (°)	95.74 (±0.0027)	96.06	95.47	94.73 ^a^, 95.62 ^b^
*V* (Å^3^)	305.93	299.63	318.43	308.24 ^a^
Density (g/cm^3^)	8.55	8.76	8.25	/

^a^ Ref. [32]; ^b^ Ref. [10].

**Table 2:** Calculated and experimental interatomic distances (Å) in SmTaO_4_.

Compound	Ta–O	Sm–O	O–O
LDA	1.9345 × 4 ^a^ 1.9955 × 4 2.2452 × 4	2.3002 × 4 2.3173 × 4 2.4774 × 4	2.6182 × 2 2.6952 × 4 2.7614 × 2 2.8789 × 4
GGA	1.9864 × 4 2.0482 × 4 2.3671 × 4	2.3570 × 4 2.3975 × 4 2.5567 × 4	2.7089 × 2 2.7779 × 4 2.8499 × 2 2.9822 × 4
Experimental	1.840 1.925 2.335	2.338 2.447 2.545	/

^a^ × 4 and × 2 mean the number of the atomic bonds.

**Table 3:** Calculated and experimental atomic coordinates in SmTaO_4_ compared with reported results.

Atom	LDA	GGA	Experimental	Reported ^a^
Sm	(0.25, 0.116, 0)	(0.25, 0.115, 0)	(0.25, 0.112, 0)	(0.25, 0.118, 0)
Ta	(0.25, 0.652, 0)	(0.25, 0.651, 0)	(0.25, 0.649, 0)	(0.25, 0.651, 0)
O1	(0.010, 0.722, 0.231)	(0.009, 0.722, 0.227)	(0.006, 0.717, 0.205)	(0.015, 0.719, 0.216)
O2	(0.909, 0.465, 0.247)	(0.911, 0.453, 0.249)	(0.894, 0.454, 0.266)	(0.901, 0.456, 0.240)

^a^ Ref. [32].

### 3.2. Electronic Structure

Since the electronic structure is crucial to understanding the optical properties of SmTaO_4_, the energy band structure and density of states (DOS) of SmTaO_4_ have been calculated with the calculated lattice constants. The GGA + *U* approach effectively describes the on-site electron-electron repulsion of the lanthanide atoms with 4*f* electrons; thus, this approach was employed in this part and was compared with LDA and GGA. The zero-point energy is set as the Fermi level.

**Figure 3:**
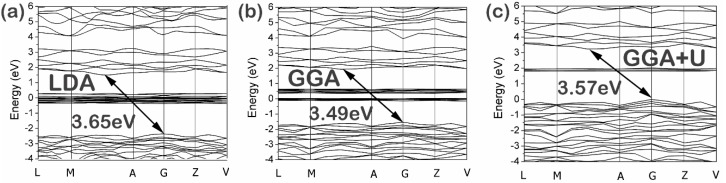
Band structure of SmTaO_4_ as calculated with (**a**) local density approximation (LDA); (**b**) generalized gradient approximation (GGA) and (**c**) GGA with additional Hubbard correlation terms (GGA + *U*)*.*

Figure 3 exhibits the energy band structure of SmTaO_4_ within the −4–6 eV region along the high-symmetry directions. The main similarity in the three images lies in the fact that the top of the valence bands appears flat, whereas the bottom of the conduction bands is slightly dispersed. In addition, a few extremely narrow bands are located in the forbidden gap that originates from the Sm 4*f* states; this outcome is consistent with the theoretical and experimental results of other lanthanide compounds [33,34]. The primary dissimilarity among the three images lies in the fact that as per the GGA + *U*, the Sm 4*f* states are observed above the Fermi level at approximately 2 eV, whereas the LDA and GGA results indicate that the Sm 4*f* states are positioned around the Fermi level. The SmTaO_4_ band gap is indirect since the valence band maxima of LDA, GGA, and GGA + *U* lie at the *G* point, whereas their conduction band minima fall between the *M* and *A* points of the Brillouin zone. The values of band gaps between the valence and conduction bands, *i.e.*, between the O 2*p* and Ta 5*d* states, are 3.65, 3.49, and 3.57 eV for LDA, GGA, and GGA + *U*, respectively; these gaps are explained in the following studies. In addition, the experimental value determined based on the diffuse reflectance spectrum is 3.68 eV. Therefore, our calculated and experimental band gap values are in agreement with the reported value of 3.8 eV (Table 4).

**Table 4:** Experimental, calculated and reported band gaps (eV) of SmTaO_4_.

Band Gap	LDA	GGA	GGA + *U*	Experiment	Reported [16]
O 2*p*–Ta 5*d*	3.65	3.49	3.57	3.68	3.8
O 2*p*–Sm 4*f*	1.35	0.98	1.76	/	/
Sm 4*f*–Ta 5*d*	0.46	0.7	1.12	/	/

**Figure 4:**
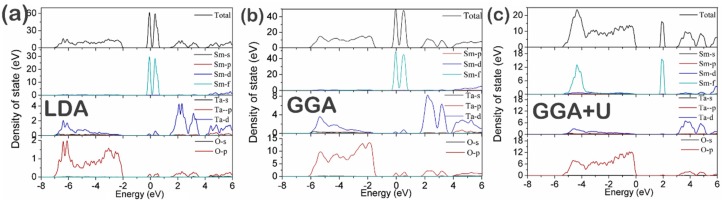
Total and partial densities of state of SmTaO_4_ as calculated with (**a**) LDA, (**b**) GGA and (**c**) GGA+ *U.*

Figure 4 shows the total density of states (TDOS) and the partial density of states (PDOS) of Sm, Ta, and O. The top of the valence band is mainly dominated by O 2*p* states hybridized with Ta 5*d* states, which suggests the contributions of covalent bonding to SmTaO_4_. The bottom of the conduction band is mainly dominated by very sharp and well-localized peaks of the Ta 5*d* states as well as slight contributions from the O 2*p* states; this phenomenon generates antibonding π^∗^ and σ^∗^ bands [35]. It can be seen from Figure 4a and Figure 4b that the LDA and GGA results show that the Sm 4*f* electrons are localized at the Fermi level and are split into two peaks. The Sm 4*f* states spread from approximately −0.45 eV to 0.80 eV for LDA and from −0.41 eV to 0.91 eV for GGA with the weak overlap of the Ta 5*d* and O 2*p* states. In the case of GGA + *U*, the Sm 4*f* states are also divided into two peaks; however, one peak is located above the Fermi level and the other is positioned at the bottom of the valence band hybridized with the Ta 5*d* and O 2*p* orbits. Table 3 summarizes the band gaps of Sm 4*f*–O 2*p* and Sm 4*f*–Ta 5*d*. The band gaps between the Sm 4*f* states at the bottom and the top of the valence band (O 2*p*) are 1.35, 0.98, and 1.76 eV for LDA, GGA, and GGA + *U*, respectively. The band gaps between the Sm 4*f* states on the top and the Ta 5*d* states at the bottom of the conduction band are 0.46, 0.70, and 1.12 eV for LDA, GGA, and GGA + *U*.

The two splitting peaks of the Sm 4*f* states are attributed to the combined effects of the spin-orbit interaction and the crystal field [14]. The splitting of the 4*f* states demonstrates the transfer of a considerable amount of charges from the O^2−^ ions to the 4*f* orbits as a result of the mixing of the O 2*p* and Sm 4*f* states. This phenomenon is also suggested by the atomic charge calculation results obtained from the Mulliken population analysis, as shown in Table 5. The atomic charges of the O atoms are −0.58 or −0.63 e (LDA), −0.63 or −0.67 e (GGA) and −0.64 or −0.68 e (GGA + *U*), while those of the Sm atoms are 1.39 e (LDA), 1.44 e (GGA) and 1.75 e (GGA + *U*).

**Table 5:** Calculated atomic charges determined by the Mulliken population analysis.

Atom	Ion	Charge (e)		
		LDA	GGA	GGA + *U*
O	1	−0.63	−0.67	−0.68
O	2	−0.58	−0.63	−0.64
O	3	−0.63	−0.67	−0.68
O	4	−0.58	−0.63	−0.64
O	5	−0.63	−0.67	−0.68
O	6	−0.58	−0.63	−0.64
O	7	−0.63	−0.67	−0.68
O	8	−0.58	−0.63	−0.64
Sm	1	1.39	1.44	1.75
Sm	2	1.39	1.44	1.75
Ta	1	1.02	1.17	0.90
Ta	2	1.02	1.17	0.90

As mentioned previously, the partially filled Sm 4*f* states are located at the Fermi level when a one-electron method with an orbital-independent potential is applied, such as LDA and GGA. This result is inconsistent with that observed in the experiments during the insulating state. In contrast, GGA + *U* facilitates the study of various strongly correlated compounds and improves considerably on LDA and GGA [36]. Therefore, GGA + *U* can provide appropriate results for SmTaO_4_.

### 3.3. Optical Properties

The complete response to the applied electromagnetic radiation field is described by the dielectric function ε(ω) = ε_1_(ω) + iε_2_(ω), where ε_1_ (ω) and ε_2_(ω) are the real and imaginary parts of the dielectric function, respectively. The intraband and interband transitions of electrons mainly contribute to ε(ω) [37]. The former are important only for metals, whereas the interband transitions can be further divided into direct and indirect transitions. The direct interband contributions to ε_2_(ω) are calculated by considering all the possible transitions from occupied to unoccupied states and taking the appropriate transition matrix element into account [38,39] as follows:

(3) $$\varepsilon_{2}\left(q\to 0,h\omega \right)=\frac{2e^{2}\pi }{\Omega \varepsilon_{0}}\sum {\left|\langle \psi_{k}^{c}\left|\hat{u},r\right|\psi_{k}^{v}\rangle \right|}^{2}\delta \left(E_{K}^{C}-E_{K}^{V}-E\right)$$

where ω is the light frequency and *e* is the electronic charge; Ψ_k_^c^ and Ψ_k_^v^ are the conduction and valence band wave functions at *k*, respectively. Index *u* is the vector defining the polarization of the incident electric field, and index Ω is the primitive cell volume. Moreover, ε_1_(ω) can be derived ε_2_(ω) through the Kramers-Kronig dispersion equation:

(4) $$\varepsilon_{1}(\omega )=1+\frac{2}{\pi }M\int_{0}^{\infty }\frac{\omega \prime \varepsilon_{2}\left(\omega \prime \right)}{\omega \prime^{2}-\omega^{2}}d\omega \prime$$

where *M* is the principal value.

Optical constants, such as refractive index *n*(ω), extinction coefficient *k*(ω), absorption coefficient *I*(ω), and reflectivity *R*(ω), can be derived from ε(ω) and are given by:

(5) $$n\left(\omega \right)={\left[\frac{\varepsilon_{1}\left(\omega \right)}{2}+\frac{\sqrt{\varepsilon_{1}^{2}\left(\omega \right)+\varepsilon_{2}^{2}\left(\omega \right)}}{2}\right]}^{1/2}$$

(6) $$k\left(\omega \right)={\left[\frac{\sqrt{\varepsilon_{1}^{2}\left(\omega \right)+\varepsilon_{2}^{2}\left(\omega \right)}}{2}-\frac{\varepsilon_{1}\left(\omega \right)}{2}\right]}^{1/2}$$

(7) $$I\left(\omega \right)=\sqrt{2}\omega {\left[\sqrt{\varepsilon_{1}^{2}\left(\omega \right)+\varepsilon_{2}^{2}\left(\omega \right)}-\varepsilon_{1}\left(\omega \right)\right]}^{1/2}$$

(8) $$R\left(\omega \right)={\left|\frac{\sqrt{\varepsilon \left(\omega \right)}-1}{\sqrt{\varepsilon \left(\omega \right)}+1}\right|}^{2}$$

**Figure 5:**
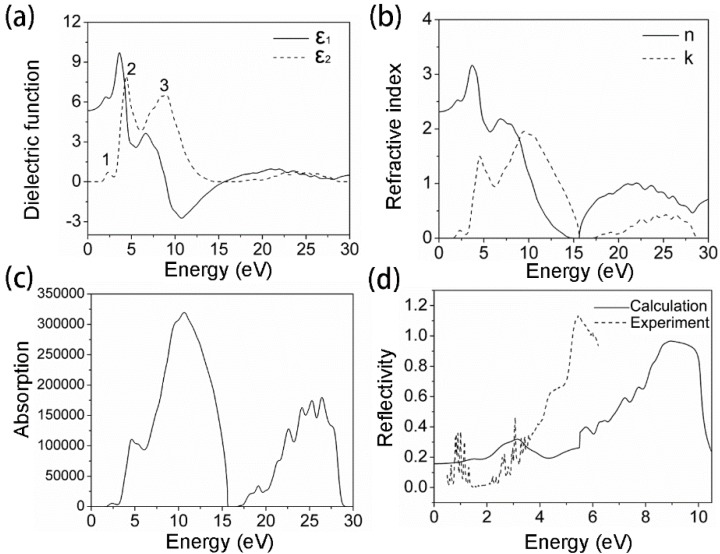
Optical properties of SmTaO_4_ as calculated with GGA + *U*: (**a**) dielectric function; (**b**) refractive index; (**c**) absorption coefficient and (**d**) reflectivity.

Since GGA + *U* accurately describes the electronic structure of SmTaO_4_, the optical property is analyzed based on the results presented by GGA + *U*. Figure 5a shows the calculated ε_2_(ω) and ε_1_(ω) of the complex dielectric function in the energy range of 0–30 eV. Three conspicuous peaks labeled 1, 2 and 3 are centered at 2.36, 4.42 and 8.83 eV, respectively; these peaks are related to the electron transitions between different special *k* points at the first irreducible Brillouin zone. Peak 1 can be ascribed to the electron transitions from O 2*p* to the Sm 4*f* states located above the Fermi level. Peak 2 is attributed to the transitions from O 2*p* to the Ta 5*d* conduction bands. Peak 3 corresponds to the transitions from the Sm 4*f* located at the bottom of the valence bands to the Ta 5*d* states. Moreover, ε_1_(ω) drops from the maximum to the local minimum in the range of 3.68–5.51 eV, and proceeds from this local maximum to another minimum in the range of 6.73–11.21 eV. This finding indicates that SmTaO_4_ has two absorption peaks in the two regions. The static dielectric constant ε(0) is approximately 5.36, as per the calculation at the equilibrium lattice constant.

Figure 5b shows the calculated refractive index. The static refractive index *n*(0) value is 2.31, which increases with increasing the energy level in the transparency region, reaching a peak in the ultraviolet region at approximately 3.70 eV. It then decreases to a global minimum at 15.03 eV. The origin of the structures in ε_2_(ω) also explains the structures in the refractive index. Furthermore, the local maxima of the extinction coefficient *k(*ω*)* correspond to the zero value of ε_1_(ω).

Figure 5c shows the behavior of the absorption coefficient *I*(ω). The absorption edge begins from approximately 1.8 eV, which corresponds to the energy band gap O 2*p*–Sm 4*f*. The first small peak located at the low-energy region originates from the electron transition from O 2*p* to the Sm 4*f* states located above the Fermi level. The subsequent two peaks in the absorption spectrum positioned at 4.62 and 10.67 eV can be explained by the dielectric function analysis results. Other peaks are observed at 19.12, 22.61, 24.13, 25.27 and 26.41 eV.

Figure 5d presents the experimental and calculated reflectivity. Due to the limitation in the experimental measurement, we merely observed the reflectivity of SmTaO_4_ at 0.5–6.2 eV. To facilitate the most accurate comparison, we conducted Savitzky-Golay smoothing by performing a local quartic polynomial regression on the experimental curve. It can be seen that the experimental reflectivity curve is reproduced properly, although the energetic positions in both spectra are not accurately consistent. This disparity is also reported in the literature [40].

## 4. Conclusions

In this work, we investigate the crystal structure, electronic structure, and optical properties of SmTaO_4_ by using an experimental method and first principles. The results show that SmTaO_4_ can be synthetized at 1400 °C, which is naturally observed with the monoclinic phase. The calculated lattice constants of SmTaO_4_ are in good agreement with the experimental values. Moreover, the electronic structure and optical properties of SmTaO_4_ were extensively studied according to three exchange-correlation potentials, namely LDA, GGA and GGA + *U*. The top of the valence band is dominated by the O 2*p* states hybridized with Ta 5*d* states, and the bottom of the conduction band is dominated by the Ta 5*d* states with slight contributions from the O 2*p* states. The two splitting peaks of the Sm 4*f* states are attributed to the combined effects of the spin-orbit interaction and the crystal field. In addition, the LDA and GGA results show that these two Sm 4*f* peaks are located around the Fermi level; in contrast, the GGA + *U* results indicate that one peak is located above the Fermi level at approximately 2 eV, whereas the other peak is positioned at the bottom of the valence band. The outcomes obtained with GGA + *U* should be more credible than those of the other two potentials because the former facilitates the study of various strongly correlated compounds and improves considerably on LDA and GGA. Furthermore, the optical properties of SmTaO_4_ are mainly composed of contributions from three types of electron transitions: the electrons from O 2*p* to the Sm 4*f* states located above the Fermi level, the electrons from O 2*p* to the Ta 5*d* conduction bands, and the electrons from the Sm 4*f* located at the bottom of valence bands to the Ta 5*d* states. The experimental reflectivity curve is reproduced properly through calculation, although the energy positions in the spectra are slightly offset.
